# A Novel RORA Hinge‐Region Variant in Adult IDDECA With Cerebellar Atrophy and Marked Response to Valproate

**DOI:** 10.1111/ene.70699

**Published:** 2026-07-12

**Authors:** V. Busco, G. Di Lazzaro, A. T. Cimmino, F. Bove, L. Saccinto, G. Silvestri, A. R. Bentivoglio

**Affiliations:** ^1^ Università Cattolica del Sacro Cuore Rome Italy; ^2^ Fondazione Policlinico Universitario A. Gemelli IRCCS Rome Italy; ^3^ R&I Genetics Padova Italy

**Keywords:** cerebellar ataxia, cerebellar atrophy, cortical myoclonus, IDDECA, intellectual disability, neurogenetics, RORA, valproate

## Abstract

**Objectives:**

To expand the *RORA* mutational and clinical spectrum by reporting a novel hinge‐region variant associated with an adult progressive phenotype and a marked therapeutic response to valproate.

**Methods:**

We describe a 61‐year‐old woman with lifelong intellectual disability who developed subacute severe motor deterioration in adulthood. Evaluation included brain MRI and whole‐exome sequencing.

**Results:**

Whole‐exome sequencing identified a novel heterozygous variant, c.551C>G (p.Pro184Arg), in the hinge region (domain D) of *RORA*, a region not previously implicated in human disease. While *RORA*‐related disorders are typically described as neurodevelopmental conditions, our patient showed progressive motor decline with loss of independent ambulation. Brain MRI demonstrated marked vermian atrophy, supporting a possible progressive component. Withdrawal of valproate was followed by severe clinical worsening, whereas reintroduction led to marked improvement in tremor and gait, restoring independent ambulation.

**Discussion:**

This case expands the mutational and phenotypic spectrum of *RORA*‐related disorders and suggests that hinge‐region variants may contribute to a more severe phenotype. It also supports a role for cortical hyperexcitability in the motor manifestations and highlights a potentially treatable component, with implications for clinical management.

## 1 Introduction

RORα (retinoic acid receptor–related orphan receptor alpha) encodes a nuclear transcription factor essential for cerebellar development [1]. Its role was first elucidated about 30 years ago by Hamilton et al., who observed that homozygous intragenic deletions of *RORa* in mice caused the “staggerer” mutant phenotype, characterized by ataxic gait and neurodegeneration of cerebellar Purkinje cells [1].

For many years, no pathogenic variants were reported in humans, until 2018, when Guissart et al. published a meta‐analysis describing heterozygous *RORA* variants in 16 individuals from 13 families [2]. The affected subjects presented variable neurodevelopmental delay, autistic traits, intellectual disability, cerebellar ataxia, and epilepsy [2]. This defined a novel autosomal dominant nosological entity known as IDDECA (*Intellectual Developmental Disorder with or without Epilepsy or Cerebellar Ataxia*). Here we report an adult case with progressive motor deterioration and marked response to valproate, highlighting a potentially treatable component of the disorder.

## 2 Case Presentation

The patient was born in 1962. Her medical history revealed febrile seizures with onset at 9 months and moderate intellectual disability. At 15 years, she developed generalized tonic–clonic seizures, successfully controlled with primidone and valproic acid. From adolescence onward, a progressive action tremor predominantly affecting the upper limbs and head was observed, worsened by emotional stress.

**FIGURE 1:**
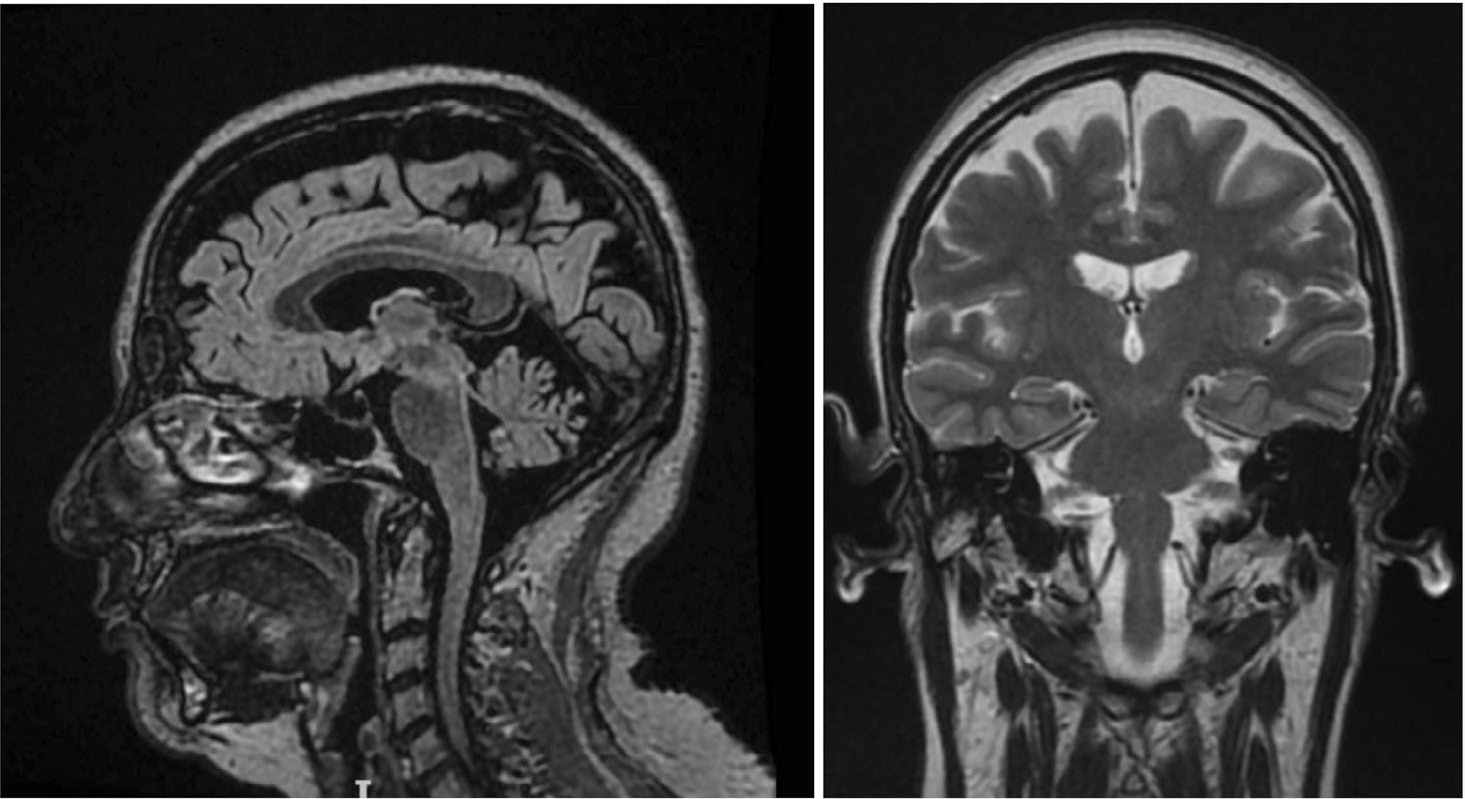
Sagittal 3D‐CUBE FLAIR and coronal T2 FSE brain MRI images showing predominant vermian cerebellar atrophy (sagittal) and enlargement of the pericerebellar and periencephalic subarachnoid spaces (coronal), suggestive of cortical and cerebellar volume loss.

In 2012, gait disturbances with dystonic posture of the right foot were treated with biperiden (4 mg/day), later discontinued due to inefficacy. Worsening of her motor symptoms led to a trial of levodopa/benserazide in February 2021, discontinued for worsening of gait. In June 2021, she was reevaluated in our epilepsy clinic: a 24‐h Holter EEG revealed no epileptic discharges. Valproic acid was withdrawn under the hypothesis of drug‐induced tremor, after which ataxia and tremor progressively worsened, and in 2022 she was hospitalized in our Neurology Department due to complete loss of independent ambulation. During hospitalization, CSF analysis (chemical and microbiological) was normal. EEG showed diffuse slowing without epileptiform activity; nerve conduction studies were normal. Ophthalmological examination was unremarkable (normal optic discs, vessels, macula, and retinal periphery). Brain MRI revealed predominant vermian cerebellar atrophy, with enlargement of the periencephalic and pericerebellar subarachnoid spaces, reflecting cortical and cerebellar volume loss (Figure 1). Given the suspicion of genetic ataxia, a *movement disorder gene panel* was performed but yielded negative results. Neuropsychological assessment indicated a mild progression of cognitive deficits beyond her intellectual disability, with a predominant disexecutive pattern consistent with early fronto‐subcortical decline. Genetic testing was then extended through *whole‐exome sequencing* targeting neurodevelopmental genes, which identified a heterozygous variant of uncertain significance, NM_134261.3:c.551C>G (p.Pro184Arg), in *RORA*. In 2023, under the hypothesis of a cortical myoclonic component contributing to tremor, valproic acid was reintroduced, leading to marked clinical improvement, in tremor and in gait. She progressively regained independent ambulation. Currently, she walks unaided indoors, with a broad‐based ataxic gait, and uses a walker for outdoor mobility (Video S1).

### 2.1 Standard Protocol Approvals, Registrations, and Patient Consents

Written informed consent for publication of clinical data, MRI images, and video material was obtained from the patient's legally authorized representative. According to local regulations, formal ethical committee approval was not required for this single case report.

## 3 Discussion and Literature Review

### 3.1 Role of RORα and Genotype–Phenotype Correlation

The RORα protein includes six functional domains. The most relevant are the C domain (DNA‐binding domain, DBD) and the E domain (ligand‐binding domain, LBD), which primarily binds cholesterol derivatives [3].

Guissart et al. identified two distinct phenotypic groups depending on the pathogenic mechanism—dominant‐negative/toxic vs. haploinsufficiency [2]. Variants within the DBD lead to aberrant DNA binding and a dominant‐negative toxic effect, resulting in epilepsy (mostly generalized), moderate to severe intellectual disability, and cerebellar vermis atrophy with ataxia [2]. Variants within the LBD cause haploinsufficiency, producing milder phenotypes, typically autism spectrum disorder with or without mild intellectual disability or epilepsy [2].

The variant detected in our patient (p.Pro184Arg) has not been previously reported. Her phenotype—vermal cerebellar atrophy, cerebellar ataxia, moderate intellectual disability, and generalized epilepsy—is consistent with the “dominant‐toxic” spectrum proposed by Guissart et al. [2].

**FIGURE 2:**
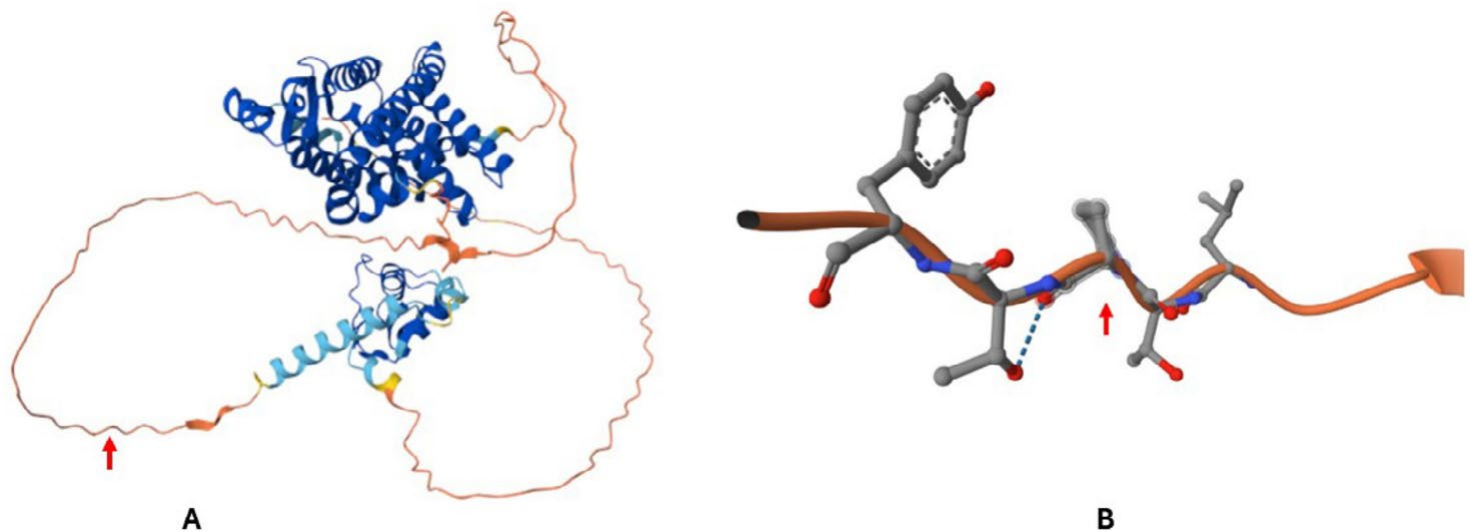
In silico structural mapping of the RORα NM_134261.3:C.551C>G, p.(Pro184Arg), variant. (A) AlphaFold‐predicted structure of RORα, showing the localization of the Pro184‐containing region, indicated by the red arrow. (B) Magnified view of the Pro184‐containing hinge region, with Pro184 indicated by the red arrow. Pro184 lies between the DNA‐binding domain and the ligand‐binding domain. The local AlphaFold confidence score at Pro184 was very low (pLDDT = 39.06), limiting the reliability of quantitative three‐dimensional structural interpretation. The p.(Pro184Arg) substitution replaces a rigid, nonpolar proline with a larger, positively charged arginine, potentially affecting local flexibility and electrostatic properties.

Residue 184 lies within the hinge region, or domain D, located between the DNA‐binding domain (DBD; aa 73–138) and the ligand‐binding domain [4]. Although Pro184 is not part of the canonical DBD, the hinge region may contribute to the spatial organization of nuclear receptors and modulate receptor–DNA interaction and transcriptional regulation [5, 6, 7, 8]. Structural mapping on the AlphaFold‐predicted RORα model showed that Pro184 falls within a region of very low local confidence (pLDDT = 39.06, Figure 2), limiting the reliability of quantitative 3D interpretation. Nevertheless, from a physicochemical perspective, the p.(Pro184Arg) substitution replaces a rigid, nonpolar proline with a larger, positively charged arginine, potentially altering local flexibility and electrostatic properties within the hinge region. Therefore, any effect on RORα DNA binding or downstream transcriptional activity remains hypothetical and requires functional validation.

Additional in silico analyses were performed. Franklin classified the NM_134261.3:c.551C>G, p.(Pro184Arg) variant as a variant of uncertain significance while indicating that the ACMG PM2 criterion was fulfilled because of its extremely low frequency in population databases [9]. Gene‐level constraint data from gnomAD showed a missense *Z*‐score of 3.41 for RORA, supporting intolerance to missense variation and providing evidence consistent with ACMG PP2. In addition, the patient's phenotype—including intellectual disability, generalized epilepsy, cerebellar ataxia, and vermian cerebellar atrophy—was highly concordant with the RORA‐related IDDECA spectrum, supporting ACMG PP4. Notably, CADD v1.7 analysis, using the GRCh38/hg38 coordinate chr15:60511495G>C, yielded a PHRED score of 23.0, placing the variant among the top 1% of potentially deleterious single‐nucleotide variants and supporting a possible functional impact. REVEL yielded an intermediate/uncertain score of 0.40. Additional predictors also suggested a possible deleterious effect, including PrimateAI (0.79; pathogenic > 0.7), MutationTaster (1.0; “disease‐causing”), FATHMM (−3.41; pathogenic <−1.5), DANN (0.94; pathogenic > 0.9), BayesDel (0.37; deleterious, moderate), and fitCons (0.72; deleterious). Overall, the variant fulfills PM2 and has additional supporting evidence consistent with PP2 and PP4, but these criteria remain insufficient to establish likely pathogenicity according to ACMG/AMP rules. Therefore, the variant remains classified as a VUS, although its rarity, phenotypic concordance, RORA missense constraint, high evolutionary conservation—including mammalian and vertebrate phastCons scores of 1.0 and vertebrate phyloP score of 11.295—and localization in a functionally relevant region of RORα support its candidacy.

The patient had no known family history of neurological disease, and both parents were reportedly neurologically unaffected. A segregation study could not be performed since the patient's parents are deceased. However, Guissart et al. reported de novo variants in 75% of cases [2], a finding confirmed in the largest published cohort, in which approximately 72.5% of variants occurred de novo [10].

### 3.2 Neuroimaging Findings

Brain MRI in RORA‐related neurodevelopmental disorder commonly shows cerebellar involvement, predominantly affecting the vermis [2, 10]. In the cohort reported by Talarico et al. 55% of individuals displayed cerebellar abnormalities, including isolated vermian hypoplasia, vermian atrophy—often superiorly predominant—and combined hypoplasia/atrophy, with serial imaging suggesting possible progression over time. Mild nonspecific supratentorial changes were observed in a minority [10]. In our patient, MRI demonstrated predominant vermian atrophy with enlargement of pericerebellar and periencephalic subarachnoid spaces, reflecting cerebellar and cortical volume loss. These findings support the vermis as a core neuroanatomical target and highlight the potential for progressive cerebellar degeneration in adults with RORA variants. Overall, the MRI pattern in our patient aligns with previously reported cases, particularly those with DNA‐binding domain variants, which are most frequently associated with cerebellar atrophy, supporting a possible progressive component beyond the neurodevelopmental phenotype [2, 10].

### 3.3 Pathophysiological Interpretation of Tremor

The dramatic response to valproate suggests that the motor impairment was primarily due to cortical myoclonus rather than cerebellar tremor alone [11, 12, 13, 14]. Given RORα's role in Purkinje cell development and maintenance, pathogenic variants may lead to Purkinje cell dysfunction and loss of cerebellar inhibitory output, resulting in increased cortical–cerebellar excitability, potentially explaining the observed cortical myoclonus and its responsiveness to valproate [1]. Valproate enhances GABAergic tone and reduces cortical excitability. Additionally, valproate inhibits histone deacetylases (HDACs), modulating neuronal gene expression—potentially relevant in disorders involving transcription factor dysfunction such as RORα‐related diseases [15].

Electrophysiological investigations to confirm the cortical origin of the movement disorder were not performed because of limited patient cooperation and refusal of further diagnostic studies; therefore, the proposed pathophysiological interpretation is mainly supported by clinical phenomenology and the marked therapeutic response to valproate.

### 3.4 Therapeutic Implications

Given the extreme rarity of RORA‐related disorders, no established treatment guidelines exist. This case suggests that valproate may represent a rational therapeutic option in RORA phenotypes associated with dominant‐toxic mechanisms.

Other agents targeting cortical hyperexcitability, such as levetiracetam or clonazepam, may be considered.

### 3.5 Limitations and Perspectives

The main limitation is the lack of functional validation in vitro or in vivo.

In vitro assays—such as reporter gene analysis, DNA‐binding capacity, protein stability, and subcellular localization studies—would help clarify whether the variant exerts a dominant‐negative effect. Knock‐in cellular or animal models could further characterize cerebellar phenotypes and Purkinje cell dendritic abnormalities. Accumulation of additional clinical cases will be essential to delineate genotype–phenotype correlations and expand the clinical spectrum of RORA‐related disorders. A further limitation is the lack of electrophysiological confirmation of cortical myoclonus.

## 4 Conclusions

This case expands the clinical spectrum of RORA‐related disorders by highlighting adult progression and suggests that cortical hyperexcitability may represent a treatable component of the phenotype, with potential therapeutic implications.

## Author Contributions

**A. T. Cimmino:** data curation, investigation. **V. Busco:** conceptualization, data curation, investigation, writing – original draft, writing – review and editing, methodology, validation, visualization, formal analysis. **L. Saccinto:** validation, formal analysis, methodology. **G. Silvestri:** writing – review and editing, validation. **F. Bove:** writing – review and editing. **G. Di Lazzaro:** conceptualization, investigation, data curation, writing – review and editing, methodology, supervision. **A. R. Bentivoglio:** conceptualization, writing – review and editing, methodology, validation, supervision.

## Ethics Statement

According to institutional policy, formal approval by an Institutional Review Board was not required for this single‐patient case report. We confirm that we have read the Journal's position on issues involved in ethical publication and affirm that this work is consistent with those guidelines.

## Consent

Written informed consent was obtained from the patient for publication of this case report and accompanying video material.

## Conflicts of Interest

The authors declare no conflicts of interest.

## Supporting information


**Video S1:** Follow‐up neurological examination. The video shows the patient during a follow‐up visit after initiation of valproate therapy. Ocular examination reveals fragmented smooth pursuit and slowed, occasionally dysmetric saccades. Gait is slightly wide‐based but autonomous, with dystonic posturing of the right upper limb and right foot. Upper limb examination demonstrates myoclonic movements of the fingers and hands during posture maintenance and voluntary movements.

## Data Availability

The data that support the findings of this study are available on request from the corresponding author. The data are not publicly available due to privacy or ethical restrictions.
